# Degraded Arabinogalactans and Their Binding Properties to Cancer-Associated Human Galectins

**DOI:** 10.3390/ijms22084058

**Published:** 2021-04-14

**Authors:** Lukas Pfeifer, Alexander Baumann, Lea Madlen Petersen, Bastian Höger, Eric Beitz, Birgit Classen

**Affiliations:** 1 Department of Pharmaceutical Biology, Pharmaceutical Institute, Christian-Albrechts-University of Kiel, 24118 Kiel, Germany; lpfeifer@pharmazie.uni-kiel.de (L.P.); abaumann@pharmazie.uni-kiel.de (A.B.); 2 Department of Pharmaceutical Chemistry, Pharmaceutical Institute, Christian-Albrechts-University of Kiel, 24118 Kiel, Germany; lpetersen@pharmazie.uni-kiel.de (L.M.P.); bhoeger@pharmazie.uni-kiel.de (B.H.); ebeitz@pharmazie.uni-kiel.de (E.B.)

**Keywords:** galectin, arabinogalactan, cell-free protein production, biolayer interferometry, *Echinacea purpurea*, *Zostera marina*

## Abstract

Galectins represent β-galactoside-binding proteins with numerous functions. Due to their role in tumor progression, human galectins-1, -3 and -7 (Gal-1, -3 and -7) are potential targets for cancer therapy. As plant derived glycans might act as galectin inhibitors, we prepared galactans by partial degradation of plant arabinogalactan-proteins. Besides commercially purchased galectins, we produced Gal-1 and -7 in a cell free system and tested binding capacities of the galectins to the galactans by biolayer-interferometry. Results for commercial and cell-free expressed galectins were comparable confirming functionality of the cell-free produced galectins. Our results revealed that galactans from *Echinacea purpurea* bind to Gal-1 and -7 with K_D_ values of 1–2 µM and to Gal-3 slightly stronger with K_D_ values between 0.36 and 0.70 µM depending on the sensor type. Galactans from the seagrass *Zostera marina* with higher branching of the galactan and higher content of uronic acids showed stronger binding to Gal-3 (0.08–0.28 µM) compared to galactan from *Echinacea*. The results contribute to knowledge on interactions between plant polysaccharides and galectins. Arabinogalactan-proteins have been identified as a new source for production of galactans with possible capability to act as galectin inhibitors.

## 1. Introduction

Galectins are a family of evolutionarily conserved glycan-binding proteins present in organisms from nematodes to mammals, which are characterized by their affinity for β-galactosides. The latter residues are present intracellularly, in the extracellular matrix or as part of cell surface glycoconjugates [1]. Although galectins do not have a signal sequence, which would be required for protein secretion through the classical secretory pathway, they can be secreted by a non-classical mechanism that bypasses the Golgi apparatus [2]. Up to now, 15 mammalian galectins have been described which share primary structural homology in their carbohydrate recognition domain (CRD, [3]). The CRDs of galectins consist of ~130 amino acids with a conserved β-galactoside binding site. Human galectins are classified into three groups according to their structure: prototypical, chimeric, and tandem repeat [4,5]. Prototypical galectins (Gal-1, -2, -5, -7, -10, -11, -13, -14, -15) contain a single CRD and can associate to form noncovalent homodimers. Gal-3 is the only mammalian representative of the second subfamily, the chimeric galectins, which have a single C-terminal CRD and a large N-terminus of about 120–160 amino acids. Association via the N-terminal domain leads to Gal-3 pentamers [6]. The tandem-repeat galectins (Gal-4, -8, -9, -12) have two different CRDs connected by a flexible peptide linker of 5 to more than 50 amino acids.

Galectins are involved in many physiological functions, e.g., immune response, inflammation, cell migration and signaling, but also in pathological processes like inflammation, fibrosis or cancer development and progression [4]. Tumor cells have the ability to create immunosuppressive microenvironments, thereby avoiding immune destruction, and galectins are key players in this process, e.g., by promotion of T cell apoptosis and inhibition of T cell activation [7]. Due to these activities in tumor immune escape, galectin expression signatures could serve as prognosis/diagnosis biomarkers [8]. Furthermore, galectins are potential targets for cancer therapy and the search for galectin inhibitors is currently in the focus of scientific interest [9]. Besides synthetic molecules, plant derived polysaccharides containing β-galactose might act as galectin inhibitors. Especially modified citrus pectins [10,11] or galactomannans [12] have been already shown to interact with Gal-3 and some are tested in clinical trials [13]. Although it seems that pectins and galactomannans bind only poorly to the carbohydrate binding sites of different galectins [14], an interaction at other binding regions of the proteins is possible [11,15]. Whereas in pectins and galactomannans the content of galactose (Gal) is relatively low, Gal is the dominating monosaccharide in a special group of plant cell wall glycoproteins, the so-called arabinogalactan-proteins (AGPs). AGPs are complex macromolecules with a relatively small protein backbone, which is covalently linked via hydroxyproline to large arabinogalactan moieties (usually around 90% of the AGP; for review see [16]). The AGP of *Echinacea purpurea* is a typical example for angiosperm land plant AGPs and mainly consists of 3-, 6- and 3,6-linked β-D-Gal residues, substituted with α-L-arabinose (Ara) and smaller amounts of β-D-glucuronic acid (GlcA) residues [17]. A recently characterized AGP of the seagrass *Zostera marina* has been found to be rich in glucuronic acids, possibly due to adaption to the marine environment [18]. AGPs have been shown to exhibit some immunomodulating activities in vitro, e.g., binding of AGPs from the medicinal plant *Echinacea purpurea* to human leucocytes [19]. The question arises, how oral uptake of macromolecular AGPs present in phytomedicines or daily food allows interaction with the human immune system. Potential binding partners of plant galactans are human galectins, which are also present in the gastrointestinal tract. Therefore, the aim of our work was to investigate the potential of galactans to bind to different galectins. Due to structural differences in the arabinogalactan part (see above), AGPs from the medicinal plant *Echinacea purpurea* and the seagrass *Zostera marina* were used as starting materials and chemically degraded to obtain saccharides of higher galactose content and lower molecular weight. The chosen galectins-1, -3 and -7 show presence in the gastrointestinal tract and/or in immune cells and have been described to be involved in cancer progression [8,20].

Besides obtaining commercial galectins, we produced Gal-1 and -7 using an *Escherichia coli* S30 extract-based cell-free transcription/translation system [21]. With this procedure, we established a rapid and efficient access route to galectins. Binding of galactans derived from AGPs to galectins was shown by biolayer interferometry. The results contribute to knowledge on sugar-binding specificities of Gal-1, -3, and -7 and broaden the understanding of interactions between plant-derived galactans and galectins.

## 2. Results

### 2.1. Chemical Characterization of Galactans

**Figure 1:**
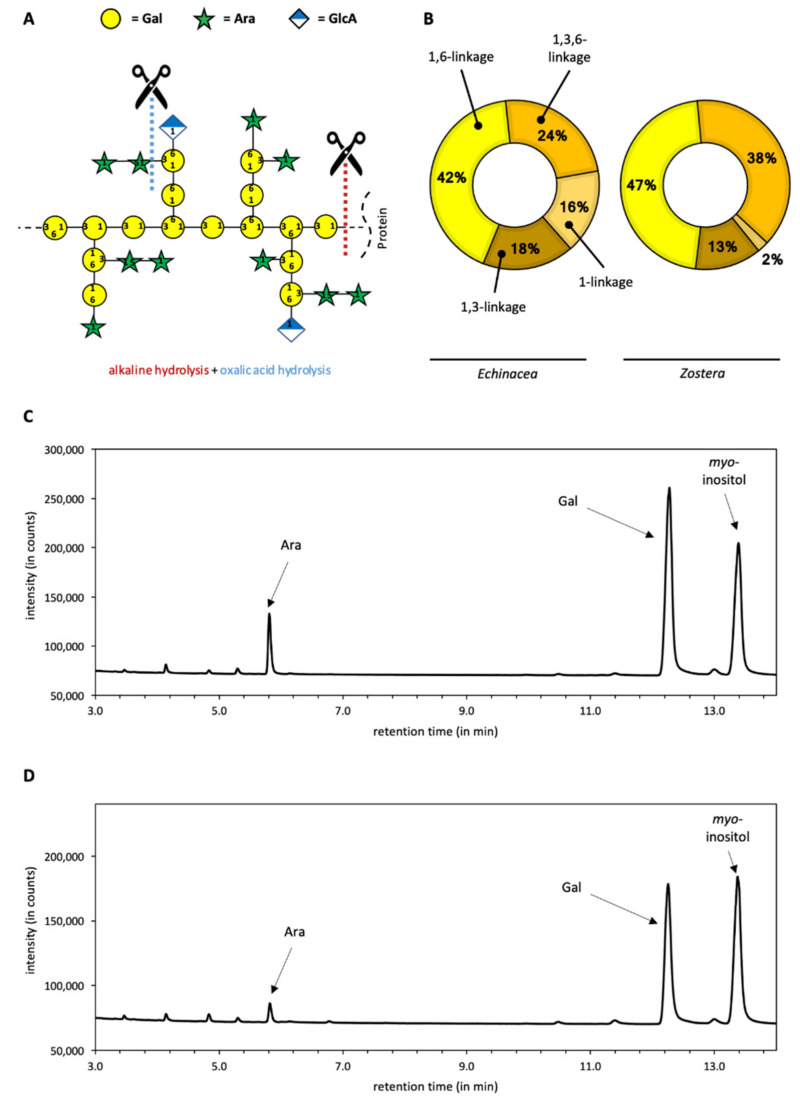
Overview on characteristics of galactans from *Echinacea* and *Zostera*. (**A**) Schematic illustration of the degradation process by alkaline and oxalic acid treatment based on a hypothetical AGP model. (**B**) Linkage-type composition of galactose part of both galactans according to [17,18]. (**C**) Gas chromatogram of the resulting derivatized monosaccharides (myo-inositol is used as internal standard) in galactans from *Echinacea*. (**D**) Gas chromatogram of the derivatized monosaccharides from *Zostera*.

Arabinogalactan-proteins were isolated from the medicinal plant *Echinacea purpurea* and from the Baltic seagrass *Zostera marina*. Both AGPs were degraded by alkaline and subsequent acid treatment (Figure 1A). Alkaline treatment leads to destruction of the protein moiety, whereas mild acid hydrolysis causes cleavage of labile monosaccharide linkage types. In case of AGPs, these are mainly furanosidic Arabinose (Ara) residues present as terminal sugars and located at the periphery of the molecules. The chemical composition of the resulting galactans is shown in Figure 1B–D.

Ara and especially Gal are the dominating monosaccharides in degraded *Echinacea* and *Zostera* AGPs (Figure 1C,D), and the Gal-linkage types typical for angiosperm AGPs are present (Figure 1B). Interestingly, branching of galactan core represented by 1,3,6-Gal is more pronounced in *Zostera* (Figure 1B). Table 1 shows the comparison of the neutral monosaccharide composition of the degraded AGPs. Both galactans consist mainly of Gal, which is present in amounts of more than 80%. The ratio of Ara to Gal is 1:5 and 1:13 in the degraded samples of *Echinacea* AGP and *Zostera* AGP, respectively. Beside these two major monosaccharides, smaller amounts of other neutral monosaccharides (fucose, glucose, mannose, rhamnose, and xylose) are present in both samples.

**Figure 2:**
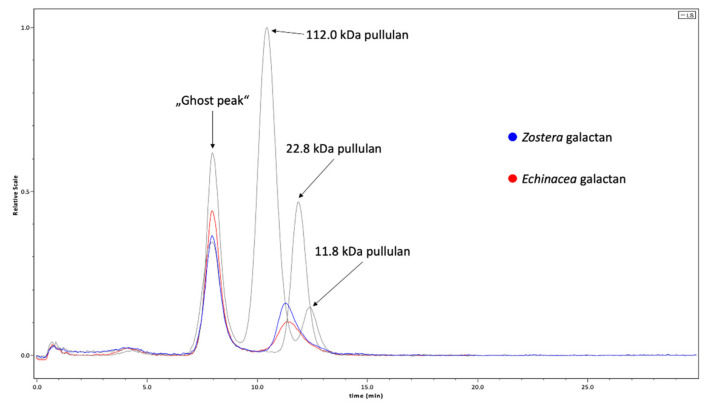
Gel-permeation chromatograms showing MALLS signals of *Zostera* galactan (blue) and *Echinacea* galactan (red) in comparison to standard pullulans with molecular weights of 112, 22.8 and 11.8 kDa. At the beginning of all runs, a ghost peak appears (explanation see text above).

The degradation process led to reduction of molecular weights of both AGPs in comparison to the original AGPs, which are characterized by molecular weights of 150–200 kDa (data not shown). In Figure 2, the results of the gel-permeation chromatography (GPC) in comparison to standard pullulans are shown. The absolute molecular weight of the galactan from *Zostera* with approximately 29.7 ± 0.8% kDa is slightly higher than the molecular weight of the galactan from *Echinacea* (approximately 25.7 kDa ± 0.7%). In both samples and also in the standards, a ghost peak appears at the exclusion volume. As investigations were performed on a borrowed column provided by the company, it is reasonable that this peak is caused by columnar bleeding.

### 2.2. Cell-Free Production of Gal-1 and Gal-7

**Figure 3:**
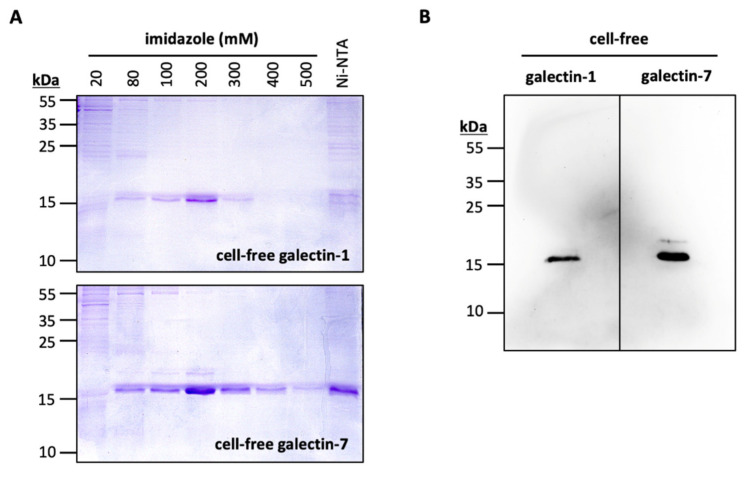
Cell-free production of Gal-1 and Gal-7. (**A**) Affinity-purification of His10-tagged Gal-1 and Gal-7 using nickel nitrilotriacetic acid agarose (Ni-NTA). Galectins were eluted stepwise by increasing imidazole concentrations. (**B**) Western blot of Gal-1 and Gal-7 (5 µg per lane) using an anti penta-His primary antibody.

N-terminally His_10_-tagged Gal-1 and Gal-7 were successfully produced using an *Escherichia coli*-based continuous exchange cell-free transcription/translation system (Figure 3). We affinity-purified Gal-1 and Gal-7 using nickel nitrilotriacetic acid agarose (Ni-NTA). Galectins were stepwise eluted using increasing imidazole concentrations in the purification buffer (Figure 3A). We confirmed the identity of Gal-1 and Gal-7, respectively, by immunoblotting (Figure 3B). With batch sizes of 4 mL cell-free reaction mixture and by pooling the elution fractions (with 80–500 mM imidazole), we obtained 0.95 mg Gal-1, and 1.5 mg Gal-7 for further use in interaction assays.

### 2.3. Binding of *Echinacea* Galactan to Gal-1 and -7 by Biolayer Interferometry (BLI)

**Figure 4:**
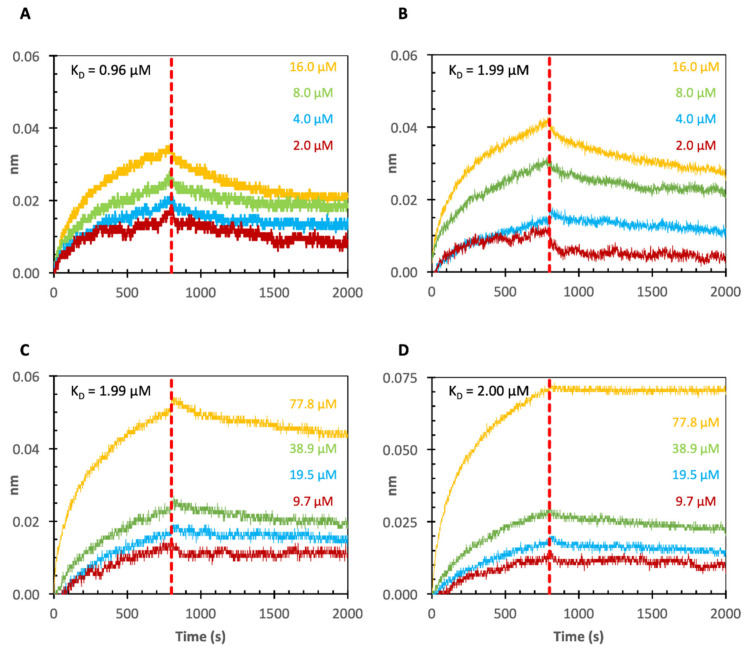
Results of BLI-experiments with *Echinacea* galactan and commercially purchased Gal-1 (**A**) and Gal-7 (**B**), as well as the corresponding cell-free produced Gal-1 (**C**) and Gal-7 (**D**).

In initial experiments (Figure 4 and Table 2), *Echinacea* galactan was tested for binding to Gal- 1 and Gal-7. To obtain information about possible galectin–AGP interactions, BLI was used. Furthermore, the cell-free expressed galectins were compared to commercially purchased Gal-1 and Gal-7 with regard to binding affinity values. Galectins were biotinylated to enable loading to streptavidin (SA) sensors. The nm-shift in the interference pattern of reflected light at the sensor surface layer is a parameter proportional to the number of bound molecules. Subsequently, the coated sensors were blocked with biocytin to inactivate unbound areas on the sensor and they were dipped into solutions with different concentrations of the galactans from *Echinacea*. The association and dissociation were monitored via nm-shifts. Fitting was best using the 1:1 binding model in the analysis software. Overall, the loading nm-shifts were quite low (0.2–0.4 nm; data not shown in Figure 3). Corresponding to this low loading, the resolution in the association and dissociation curves of the sensograms (Figure 4A–D) was also quite low. Despite of this, all samples showed good concentration dependent binding with K_D_ values of 1–2 µM. The calculation of K_D_-values for Gal-1 and -7 showed nearly identical values for the commercially purchased and the cell-free produced galectins (Figure 4 and Table 2), underlining that cell-free production of Gal-1 and Gal-7 was successful and preserved functionality of these proteins. Furthermore, it was shown that K_D_-values for binding of *Echinacea* galactan to Gal-1 and Gal-7 were comparable (Figure 4 and Table 2).

### 2.4. Binding of *Echinacea* Galactan to Gal-3 by BLI

In the next experiments, we investigated binding of *Echinacea* galactan to commercially purchased Gal-3. As loading of the biotinylated Gal-1 and -7 to the sensors was quite low (see above), we compared streptavidin sensors (SAs) with super-streptavidin sensors (SSAs) in this experiment.

**Figure 5:**
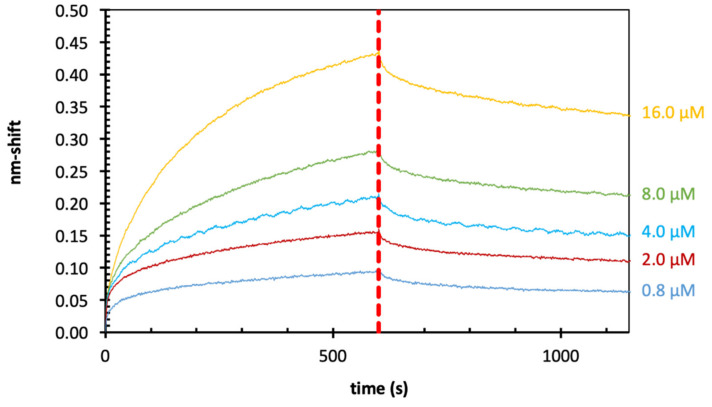
Results of BLI-experiments of *Echinacea* galactan to Gal-3 (super-streptavidin sensors).

In all experiments, high loading of biotinylated Gal-3 to the sensors was observed with nm-shift between 1.2 and 3.0 nm (data not shown in Figure 5). Loading of biotinylated Gal-3 to SSAs (2.0–2.5) was slightly better compared to loading to SAs (1.0–2.0). For both sensor types, concentration-dependent binding of *Echinacea* galactan to Gal-3 was shown (see Figure 5 for SSAs). Average K_D_-values were comparable and slightly lower with SSAs (Table 3).

### 2.5. Binding of *Zostera* Galactan to Gal-3 by BLI

**Figure 6:**
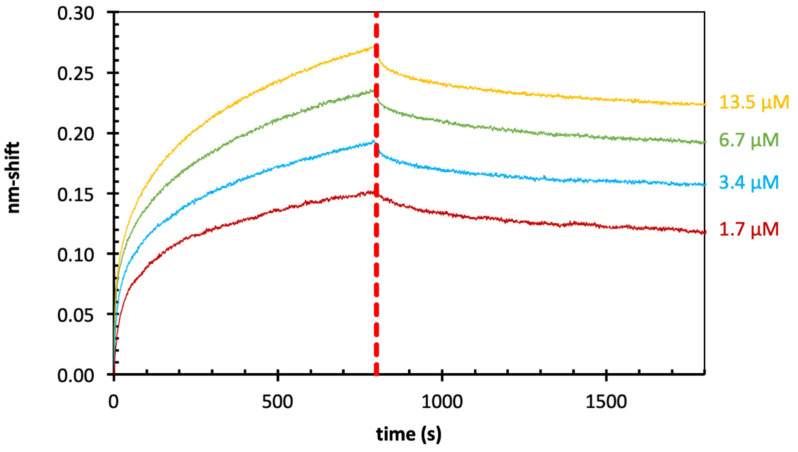
Results of BLI-experiments of *Zostera* galactan to Gal-3 (streptavidin sensors).

Finally, the ability of Gal-3 to bind galactan isolated from the Baltic seagrass *Zostera marina* with higher galactose content (Table 1) was evaluated. The overall sensogram shape (Figure 6) and range was comparable to the results shown for *Echinacea*. The calculated K_D_ binding values for *Zostera* galactan were in the range of 0.08–0.28 µM (Table 4). This means that the obtained values are slightly lower and the binding properties are slightly stronger for *Zostera* galactan than for *Echinacea* galactan.

## 3. Discussion

### 3.1. Composition of Galactans

Up to now, mainly pectins, especially from *Citrus*, and their degradation products [10,11,22] as well as galactomannans [12,23] have been tested for their binding potential to human galectins. In the pectic polysaccharides—namely homogalacturonan, xylogalacturonan, rhamnogalacturonan I, and rhamnogalacturonan II—galacturonic acid and rhamnose are the dominant monosaccharides. Galactose is present only as side chains of rhamnogalacturonan I, mainly in form of linear ββ-1,4-galactans [10]. Galactomannans are typical components of Fabaceae seeds with a backbone of β-1,4-linked mannose, some of them branched at position 6 to a galactose residue [24]. In contrast to pectins and galactomannans with relatively low Gal contents, Gal is the dominating monosaccharide in AGPs. We therefore used AGPs from *Echinacea purpurea* and the seagrass *Zostera marina* [18] as starting material for partial degradation to galactans with high galactan content and reduced molecular weight. Both products consist mainly of galactose in 1,3-, 1,6 and 1,3,6-linkage, but differ with regard to branching represented by 1,3,6-galactose which is more prominent in *Zostera* galactan.

### 3.2. Cell-Free Production of Gal-1 and -7

*E. coli* is an established expression system for recombinant human galectins [25,26,27,28], which has also been used for the production of the commercially obtained Gal-1 and Gal-7. With this work, we extended the access routes to an *E. coli* extract-based cell-free system for rapid and efficient production of human galectins suitable for functional studies. The DTT-rich cell-free reaction mix is favorable to galectins, whose functional integrity requires a reduced state [29,30]. The nearly identical affinity data obtained with the cell-based and cell-free produced Gal-1 and Gal-7 proteins indicate proper folding and imply that other galectins may be equally amenable to cell-free production as well.

### 3.3. Binding of Galactans to Galectins

#### 3.3.1. Binding of Plant Saccharides to Gal-1, -3 and -7

The chosen galectins of the different structural groups are present in the gastrointestinal tract and/or in immune cells and have been described to be involved in cancer [8]. Especially for Gal-3, interaction with different plant poly- or oligosaccharides and an effect on metastasis associated processes of cancer cells has already been proven [31].

BLI is an effective method to measure molecular interactions between one partner immobilized onto a biosensor surface and the other binding partner kept in solution. For binding of pectin-derived polysaccharides to Gal-3, general comparability of BLI to surface plasmon resonance (SPR) measurements has been shown [32]. On the other hand, even within BLI experiments, differences caused by use of different sensors (e.g., Ni-NTA sensors and streptavidin sensors) have been reported [32]. Our own results also showed small variations of K_D_ values by using streptavidin- or super-streptavidin sensors (Table 3 and Table 5) and even between repetitions of the same experiment (Table 4). Table 5 offers an overview on literature data for binding of different saccharides to Gal-3 determined by BLI in comparison to our results. Whereas binding affinities of the two disaccharides Galβ1–3/4 GlcNAc are in the micromolar range [33], different polysaccharides derived from pectin reveal stronger binding. Within the pectin derivatives, the homogalacturonans have higher K_D_ values in the micromolar range compared to the rhamnogalacturonans with K_D_ values in the nanomolar range [11,32,34,35]. Whereas HGs mainly consist of GalA, the latter comprise also galactan side chains. A rhamnogalacturonan from Ginseng binds to Gal-3 via its multiple galactan side chains [36], and galactans have been shown to be a structural element in pectin that directly binds Gal-3 [37,38].

Binding properties of the galactans from AGPs of *Echinacea* and *Zostera* to Gal-3 are also in the nanomolar range (between 80 and 700 nm), comparable to the rhamnogalacturonans, although different linkage types of Gal are present in both groups. Gal side chains of rhamnogalacturonan I are mainly β-1,4-linked, whereas the AGP-derivatives include β-1,3, β-1,6 and β-1,3,6 galactan linkage types. Of note is that the poly-β-galactosyl epitope (Galβ1–3)_n_ found on the parasite *Leishmania* is also recognized by Gal-3 [39]. The *Echinacea* galactan produced by TFA hydrolysis has previously been shown to inhibit adhesion of pancreatic carcinoma cells to liver endothelial cells in a Gal-3 dependent manner [40]. An arabinogalactan from *Panax notoginseng* inhibits pancreatic cancer cell growth [31]. In our study, binding of *Zostera galactan* to Gal-3 (80–280 nM) is slightly stronger compared to *Echinacea* galactan (360–700 nM). One difference between both is that 1,6-linked Gal and branching represented by 1,3,6-Gal is more pronounced in *Zostera* (Figure 1B). Furthermore, the content of glucuronic acid is much higher in *Zostera* AGP (around 15%, [18]) compared to *Echinacea* AGP (4–5%, [17]).

Whereas interaction of plant polysaccharides with Gal-3 has been investigated by different authors (e.g., [10,11,12,31]), binding of plant products to Gal-1 and Gal-7 has been studied rarely [23,41]. *Echinacea* galactan binds to Gal-1 and Gal-7 in a range between 1 and 2 µM, which is slightly weaker compared to binding to Gal-3. The minimal glycan ligand for mammalian galectins is the disaccharide lactosamine, Galβ1-4GlcNac, and biolayer interference studies with the two ligands Galβ1-4GlcNac and Galβ1-3GlcNac revealed, that Gal-1 and Gal-3 preferentially bound to Galβ1-4GlcNac, while binding of Gal-7 was stronger to Galβ1-3GlcNac [33]. Despite high structural homology of the CRD of all galectin members, the mode of how each galectin displays different sugar-binding specificity still remains ambiguous.

#### 3.3.2. Mode of Binding of Plant Saccharides to Galectins

Structurally, all galectin CRDs have a β-sandwich folded conformation with a six-stranded β-sheet for the canonical sugar binding S-face and an opposing five-stranded β-sheet F-face [42]. Although it seems that pectins and galactomannans bind only poorly to the canonical carbohydrate binding sites of different galectins [14], an interaction at other regions of the galectins is possible [11,15]. According to Zhang and colleagues [11], interaction of pectic polysaccharides primarily occurs with the F-face of the CRD of Gal-3, and a synergistic binding mode for HG and RG has been proposed. Additionally, for galactomannans from Guar gum, binding to the F-face seems likely [15]. A rhamnogalacturonan from Ginseng binds to the CRD, but also to the N-terminal tail of Gal-3. In that study, the galactan side chains have been proposed to act as responsible moieties [43]. Our results introduce plant arabinogalactan-proteins as new source to produce galactans capable to interact with different galectins. Binding features of these galactans have to be elucidated in the future. Knowledge on new glycan structures binding to galectins is a step forward to development of therapeutic galectin inhibitors from natural sources. Galectins impact also important malignancy-associated processes [44], such as cell growth and adhesion along with its role in modulation of immune responses. Therefore, those glycan structures with high water solubility and safe features in humans might be model substances for development of drugs in the treatment of cancer diseases. A major challenge will be the development of galectin inhibitors selective for a specific galectin.

## 4. Materials and Methods

### 4.1. Isolation of AGPs

Isolation of AGPs from *Echinacea purpurea* and *Zostera marina* was performed by Yariv precipitation as described in [17,18].

### 4.2. Partial Degradation of AGPs

For alkaline hydrolysis the isolated AGPs from *Echinacea* and *Zostera* were dissolved in 0.44 M sodium hydroxide solution in a screw-cap tube (Pyrotube C vial; Associates of Cape Cod Inc., East Falmouth, MA, USA) resulting in a final concentration of 6.7 mg/mL. The solutions were hydrolyzed for 20 h at 105 °C in a heating block (Bioblock Scientific, Thermolyne Corp., Ramsey, MN, USA). Afterwards the solution was neutralized with diluted hydrochloric acid and added to the four-fold volume of absolute ethanol and cooled overnight at 4 °C. Subsequently the precipitate was separated by centrifugation at 19,000× *g* for 30 min (Hereus Multifuge X3, Thermo Fisher Scientific Corp., Waltham, MA, USA) and additional washing with 80% (*v/v*) ethanol two times. The washed residue was redissolved in double-distilled water and freeze-dried (Christ Alpha 1–4 LSC, Martin Christ GmbH, Osterode, Germany).

A total of 20 mg of the dried sample was dissolved in 2 mL of 12.5 mM oxalic acid in a screw-cap tube and hydrolyzed for 5 h at 100 °C in the heating block. Precipitation and further treatment were performed as stated above.

### 4.3. Determination of Neutral Monosaccharide Composition

Neutral monosaccharide composition was determined according to [45] with slight modifications (see [18]). Following the acetylation and reduction, the dichloromethane layer was injected into the gas chromatograph (Agilent 7890B, Agilent Technologies column: Optima-225, 25 M, 0.25 mM, 0.25 µM; flow rate: 1 mL/min; temperature 230 °C; split ratio 30:1, Santa Clara, CA, USA).

### 4.4. Determination of Absolute Molecular Weight

An Äkta pure 25 chromatography system (GE Healthcare Bio-Sciences, Marlborough, MA, USA) coupled with a multi-angle light scattering detector (DAWN8+, Wyatt Technology Corporation, Santa Barbara, CA, USA) and an RI-detector (Optilab T-rEX, Wyatt Technology Corporation, Santa Barbara, CA, USA) was used for determination of absolute molecular mass of the galactans. These were dissolved in elution buffer (0.15 M NaCl, 0.05 M phosphate buffer, pH 7.0) and filtered through a sterile filter (Rotilabo PVDF syringe filter, 0.22 µm pore-size, Roth GmbH & CO.KG, Karlsruhe, Germany) prior to injection of 100 µL. The whole system including the Shodex OHpak SB-804 column (Showa Denko AG, Tokyo, Japan) was equilibrated with the same buffer before injection and elution (flow rate was 0.7 mL/min). Molecular weight calculation was performed with the Astra analysis software (v7.1.2, Wyatt Technology Corp.). Pullulan standards (112.0 kDa, 22.8 kDa, 11.8 kDa; PL polysaccharide standard kit SAC-10, Varian Inc., Palo Alto, CA, USA) were investigated likewise and shown in Figure 2.

### 4.5. Commercially Purchased Galectins

For the binding experiments with galectin-3 (Gal-3, human, recombinant, Sigma-Aldrich Corporation, Taufkirchen, Germany), as well as for the comparative experiments with Gal-1 (human, recombinant, Thermo Fisher Scientific Corporation, Waltham, MA, USA) and Gal-7 (human, recombinant, Sigma-Aldrich Corp.), commercially purchased galectins were used.

### 4.6. Cell-Free Production, Purification and Western Blotting of Gal-1 and Gal-7

Gal-1 and -7 were produced using a continuous exchange cell-free transcription/translation system (CECF system) based on an S30 ribosomal extract prepared from the *Eschericia coli* strain BL21(DE3) and T7 transcription [21]. The respective open reading frames including N-terminal His_10_-tags in pET16b were custom synthesized (GenScript, Leiden, Netherlands). CECF production was done as described earlier without addition of detergents [46]. Briefly, 1 mL of reaction mix containing 23 µg of plasmid DNA was injected into a dialysis cassette (SlideALyzer^®^, 10k MWCO; Thermo Fisher Scientific) and placed in a reservoir of 17 mL of feeding mix. The reactors were gently shaken in a water bath at 30 °C for 24 h. For affinity purification, 300 µL Ni-NTA (Qiagen, Hilden, Germany) and 5 mL purification buffer (20 mM HEPES, 300 mM NaCl, 10% glycerol, pH 8.0) were added per mL of cell-free reaction. The mixture was incubated overnight rotating at 4 °C and transferred to gravity flow Poly-prep^®^ chromatography columns (2 mL bed volume; Bio-Rad Laboratories, Feldkirchen, Germany). The samples were washed with 20 bed volumes of purification buffer supplemented with 20 mM imidazole, and stepwise eluted in fractions containing 80–500 mM imidazole. Elution fractions containing galectin protein were pooled and concentrated (Amicon^®^ Ultra-15 3k MWCO, Merck). For Western blotting, protein samples were separated by 15% SDS-PAGE and transferred to polyvinylidene fluoride membranes (Amersham Hybond-P 0.45, GE Healthcare Life Sciences, München, Germany) for ECL chemiluminescence detection (Bio-Rad, Feldkirchen, Germany) using a mouse anti penta-His antibody (1:5000, Qiagen, cat. no. 34660) and a horseradish peroxidase coupled secondary goat anti-mouse secondary antibody (1:5000; Jackson ImmunoResearch/Dianova, cat. no. 115-035-174, Hamburg Germany).

### 4.7. Biotinylation of Galectins

A total of 50 µg of the commercially purchased or the cell-free expressed galectins were diluted with phosphare-buffered saline (PBS: 137 mM NaCl, 2.7 mM KCl, 8.1 mM Na_2_HPO_4_, 1.8 mM KH_2_PO_4_, pH 7.4) to solutions with a concentration of 1 mg/mL. These were subjected to the biotinylation procedure following the Forté Bio Technical Note 28. Biotin (NHS-Biotin-PEG_4_, EZ-Link, Thermo Fisher Scientific Corp.) was prepared as a 1 mM solution in sterilized water and was added to the galectins in a volume derived from the following equation:

(1) $$µL\;1\;\mathrm{mM}\;\mathrm{biotin}\;\mathrm{reagent}\;=\frac{\mathrm{Protein}\;\mathrm{concentration}\;\left(\mathrm{in}\frac{\mathrm{mg}}{\mathrm{mL}}\right)}{\mathrm{molecular}\;\mathrm{weight}\;\mathrm{Protein}\;\left(\mathrm{in}\;\mathrm{kDa}\right)}\times \mathrm{MCR}\times \mathrm{volume}\;\mathrm{protein}\left(\mathrm{in}\;µL\right)$$

The molecular coupling ratio of the biotin reagent was chosen to be MCR = 1, according to the manufacturer’s recommendations. After incubation for 2 h on wet ice the unbound reagent was removed with a desalting column (Zeba Spin desalting column, 0.5 mL, Thermo Fisher Scientific Corp.) and the biotinylated galectin was immediately used for BLI.

### 4.8. BLI

For molecular binding experiments the biolayer-interferometric system Octet RED96e (FortéBio, Sartorius AG, Göttingen, Germany) was used with Dip & Read SA and SSA sensors (streptavidin and super-streptavidin, FortéBio). The sensors were soaked prior to the experimental run in PBS for 1800 s in the micro-well plate (Greiner Bio-One GmbH, Kremsmünster, Austria) of the instrument. Two 600 seconds washing steps were performed in PBS followed by a 1200 s baseline acquisition. Galectins were loaded by dipping the sensors in a 10 µg/mL solution of the respective galectin for 2000 s. Unbound sites on the sensors were covered by application of a biocytin blocking solution (10 µg/mL in PBS, EZ-Link, Thermo Fisher Scientific Corp.) for 300 s followed by 600 s of washing in PBS. A second baseline acquisition step was performed, and the sensors were dipped into galactan solutions in PBS. A range of different concentrations from 0.8 to 77.8 µM was used and one control sensor was dipped into a solution of one of the highest concentrations. In these solutions, the association was recorded for 800 s (for SSA sensors: 600 s). Afterwards the sensors were dipped into buffer solution for 1200 s to investigate the dissociation. All steps were performed under constant shaking of the plate at 1000 rpm and controlled temperature of 25 °C. For all sensors, the K_D_ values were calculated using the internal 1:1 binding model of analysis software (Octett system data analysis software, version 10.0.1.6, Forté Bio). For the commercially purchased galectins, the measurement was done two to three times with an array of concentrations. The cell-free expressed galectins were measured one time with four concentrations. Final calculation of average K_D_ value was performed with all sensors resulting in a fitting curve of R^2^ > 0.8.

## Figures and Tables

**Table 1:** Neutral monosaccharide composition of galactans. Values are given in % (mol/mol).

Monosaccharide	*Echinacea* Galactan	*Zostera* Galactan
Galactose	80.7	86.4
Arabinose	16.1	6.8
Others	3.2	6.8

**Table 2:** Binding parameters of Gal-1 and Gal-7 with *Echinacea* galactan determined by BLI. Average K_D_-values were calculated from three independent experimental runs (Gal-1 and Gal-7) or the four concentrations of one experimental run (cell-free expressed Gal-1 and Gal-7).

Sample	Binding Partner	Average K_D_-Value
*Echinacea* galactan	Gal-1	0.96 µM
*Echinacea* galactan	Gal-1 Cell-free produced	1.99 µM
*Echinacea* galactan	Gal-7	1.99 µM
*Echinacea* galactan	Gal-7 Cell-free produced	2.00 µM

**Table 3:** Binding parameters of Gal-3 with *Echinacea* galactan determined by BLI. Average values are calculated from two independent experimental runs.

Sample	Binding Partner	Sensor	Average KD-Value
*Echinacea* galactan	Gal-3	Super- streptavidin	0.36 µM
*Echinacea* galactan	Gal-3	Streptavidin	0.70 µM

**Table 4:** Binding parameters of Gal-3 with *Zostera* galactan determined by BLI. The average K_D_-values are calculated from the results of the four different concentrations for each experimental run.

Sample	Binding Partner	Sensor	Average K_D_-Value
*Zostera* galactan	Gal-3	Streptavidin	0.08 µM
*Zostera* galactan	Gal-3	Streptavidin	0.28 µM
*Zostera* galactan	Gal-3	Streptavidin	0.17 µM

**Table 5:** Overview on K_D_-values for binding of different saccharides to Gal-3 determined by BLI.

Saccharide		K_D_-Values	Sensor-Type	Reference
Disaccharides	Galβ1-3GlcNAc	0.23 µM	Super-streptavidin	[33]
	Galβ1-4GlcNAc	0.28 µM		
Pectin-derived	1,4-β-D-galactan	0.60 µM	Streptavidin	[32]
	RGI-4	0.10 µM		
	MCP	15 µM		
	RG	0.05 µM	Streptavidin	[11]
	HG1	46 µM		
	HG2	138 µM		
	RGI	0.07 nM	Ni-NTA	[34]
	RG-I (WGFPA-1a)	4.90 µM	Ni-NTA	[35]
	RG-I (WGFPA-1b)	0.24 µM		
	RG-I (WGFPA-1c)	0.71 µM		
AGP-derived	Galactan from *Echinacea*	0.70 µM	Streptavidin	This work
	Galactan from *Echinacea*	0.36 µM	Super-streptavidin	
	Galactan from *Zostera*	0.08–0.28 µM	Streptavidin	

HG: homogalacturonan; MCP: modified citrus pectin, RG: rhamnogalacturonan.
